# Swedish tobacco policy: Key learnings to decrease smoking and challenges that still lie ahead

**DOI:** 10.18332/tpc/196350

**Published:** 2024-12-20

**Authors:** Lisa L. Ermann, Lisa Klefbom

**Affiliations:** 1 Swedish Cancer Society, Stockholm, Sweden

**Keywords:** smoke-free, smoking prevalence, snus, tobacco policy, nicotine pouches, nicotine snus

Sweden has reduced tobacco smoking through tobacco policy measures (Figure 1).

**Figure 1:**
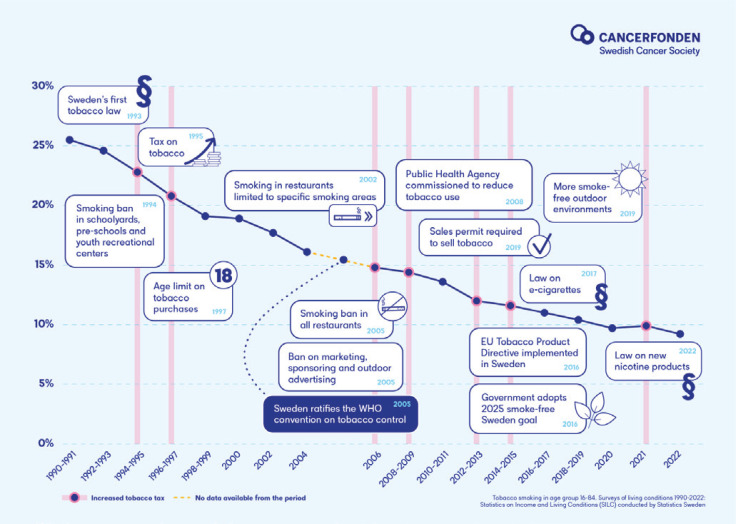
Policy measures to reduce tobacco smoking in Sweden 1990–2022

During the 1990s, Sweden implemented powerful measures that had discouraging and normative effects, stemming from the first regulations in the 1960s. Sweden was one of the first countries to introduce smoke-free workplaces^1^. Since then, several public environments have been made smoke-free, such as sport arenas, bus stops, restaurants and bars both indoors and outdoors^2^. A ban on tobacco advertising was implemented as well as an age limit, a sales permit requirement for selling tobacco was introduced and was paired with an active tax policy. These laws have been monitored and enforced by authorities, and tobacco use has been monitored continuously^3^. Smokers have been offered cost-free smoking cessation assistance since 1998 through the national Quitline as well as through primary care^4^.

Sweden is, however, not smoke free. Smoking still causes most preventable cancer cases in Sweden every year^5^. Smoking prevalence is at 11%, of which 5% is daily use. The socioeconomic differences are significant. Among people who have finished compulsory school, 16% smoke, of which 11% smoke daily. Among people with a college or university degree, 8% smoke, of which 3% smoke daily^6^.

While smoking rates have been decreasing over time, prevalence of snus use has been stable at around 11–13% daily use until 2021. However, snus use has started to increase with daily use now at 16%, driven mainly by the increase in use among young people and women. Among the group aged 16–29 years, the percentage of women who use snus daily increased from 3% in 2018 to 18% in 2024, with total current use in 2024 at 25% for women and 29% for men^6^. Among girls aged 15 years, current snus use increased from 3% in 2019 to 14% in 2024, following the same pattern^7^.

Snus is not a cessation product. On the contrary, people who use e-cigarettes or snus, are more likely to start smoking over time, compared to people who do not use e-cigarettes or snus^8^.

Regulation of different tobacco and nicotine products differ greatly, which is why the trends in cigarette use and the use of other products differ so much. The taxation of nicotine is much lower compared to tobacco taxation, making nicotine snus more affordable to cost sensitive groups^9,10^. There is no limit to nicotine content in snus. There are no regulations of flavors in e-cigarettes or snus^1,11^. Nicotine snus is the only (tobacco or nicotine) product that can be marketed, and there are major shortcomings in legal compliance with marketing regulation of modesty and intrusiveness^1,11,12^.

The total tobacco and nicotine use in Sweden is at 28% and currently increasing, including cigarette use^6,7^. It is common to use more than one tobacco or nicotine product, even among young users. Among young people (15 years) who use snus or e-cigarettes, it is more than four times more common to also smoke cigarettes, compared to young people in general^13^.

Sweden has decreased its smoking rates through decisive tobacco policy measures. Right now, Sweden has very diverse regulations and taxations on different tobacco and nicotine products. Using tobacco or nicotine products increases the risk of smoking cigarettes. Sweden is currently seeing a rapid increase of tobacco and nicotine use among the general public, particularly amongst young people. To reduce tobacco and nicotine use, all products need to be regulated equally.

## Data Availability

Data sharing is not applicable to this article as no new data were created.
